# Occurrence of and Reasons for “Missing Events” in Mobile Dietary Assessments: Results From Three Event-Based Ecological Momentary Assessment Studies

**DOI:** 10.2196/15430

**Published:** 2020-10-14

**Authors:** Katrin Ziesemer, Laura Maria König, Carol Jo Boushey, Karoline Villinger, Deborah Ronja Wahl, Simon Butscher, Jens Müller, Harald Reiterer, Harald Thomas Schupp, Britta Renner

**Affiliations:** 1 Psychological Assessment & Health Psychology Department of Psychology University of Konstanz Konstanz Germany; 2 University of Hawaii Cancer Center University of Hawaii Honolulu, HI United States; 3 Human-Computer Interaction Group Department of Computer and Information Science University of Konstanz Konstanz Germany; 4 General Psychology Department of Psychology University of Konstanz Konstanz Germany

**Keywords:** dietary assessment, diet records, mobile phone, mobile applications, technology, adherence, compliance, missing events, Ecological Momentary Assessment, mHealth

## Abstract

**Background:**

Establishing a methodology for assessing nutritional behavior comprehensively and accurately poses a great challenge. Mobile technologies such as mobile image-based food recording apps enable eating events to be assessed in the moment in real time, thereby reducing memory biases inherent in retrospective food records. However, users might find it challenging to take images of the food they consume at every eating event over an extended period, which might lead to incomplete records of eating events (*missing events*).

**Objective:**

Analyzing data from 3 studies that used mobile image-based food recording apps and varied in their technical *enrichment*, this study aims to assess how often eating events (meals and snacks) were missed over a period of 8 days in a naturalistic setting by comparing the number of recorded events with the number of normative expected events, over time, and with recollections of missing events.

**Methods:**

Participants in 3 event-based Ecological Momentary Assessment (EMA) studies using mobile image-based dietary assessments were asked to record all eating events (study 1, N=38, 1070 eating events; study 2, N=35, 934 eating events; study 3, N=110, 3469 eating events). Study 1 used a *basic* app; study 2 included 1 fixed reminder and the possibility to add meals after the actual eating events occurred instead of in the moment (*addendum*); and study 3 included 2 fixed reminders, an addendum feature, and the option to record skipped meals. The number of recalled missed events and their reasons were assessed by semistructured interviews after the EMA period (studies 1 and 2) and daily questionnaires (study 3).

**Results:**

Overall, 183 participants reported 5473 eating events. Although the momentary adherence rate as indexed by a comparison with normative expected events was generally high across all 3 studies, a differential pattern of results emerged with a higher rate of logged meals in the more technically intensive study 3. Multilevel models for the logging trajectories of reported meals in all 3 studies showed a significant, albeit small, decline over time (b=−.11 to −.14, *P*s<.001, pseudo-R²=0.04-0.06), mainly because of a drop in reported snacks between days 1 and 2. Intraclass coefficients indicated that 38% or less of the observed variance was because of individual differences. The most common reasons for missing events were competing activities and technical issues, whereas situational barriers were less important.

**Conclusions:**

Three different indicators (normative, time stability, and recalled missing events) consistently indicated missing events. However, given the intensive nature of diet EMA protocols, the effect sizes were rather small and the logging trajectories over time were remarkably stable. Moreover, the individual’s actual state and context seemed to exert a greater influence on adherence rates than stable individual differences, which emphasizes the need for a more nuanced understanding of the factors that affect momentary adherence.

## Introduction

*Eating* may seem to be one of the simplest behaviors, yet it is quite complex [1-3], involving up to 200 decisions a day [4]. Although methodological challenges exist in accurately measuring food intake in community dwellings [5,6], analyzing data on food intake is important for our understanding of diet-disease relationships and the refinement of nutrition guidelines [7], which provide essential information for surveillance, planning interventions, and policy [8].

The limitations associated with common measuring methods, such as self-reported food intake (eg, dietary records, 24-hour dietary recall, and food frequency questionnaires), which include memory biases and other measurement errors, have already been outlined [5,9-15]. However, new mobile technologies have the potential to reduce the burden on both researchers and participants by improving adherence and communication, automating and standardizing coding, and upgrading data quality ([16]; for an overview see study by Boushey et al and Eldridge et al [5,17]). Specifically, taking images of eating events has been proposed as a method for reducing the burden on participants in dietary studies. Various models based on image technology using mobile apps have been developed in recent years (eg, Technology Assisted Dietary Assessment, My Meal Mate, and SMARTFOOD; for more details, see study by Boushey et al [5], Eldridge et al [7], Villinger et al [18], and Wahl et al [2]), and they are increasingly being used to assess and change eating behavior and food intake in different populations, including patients and generally healthy adults or adolescents [19,20]. As with any dietary assessment method, the more eating events are captured, the more thorough the analysis, and in an ideal study, the user would capture all eating events including an image before and, if applicable, after consumption. Therefore, adherence during the study period is key to a valid assessment of the actual eating behavior.

In previous research, the focus was mainly on general adherence rates, such as dropout rates indexed by the number of participants who did not complete the whole study period, or missing value rates for study variables indexed by the number of participants who did not respond to some of the study variables. For example, a smartphone electronic food diary app called My Meal Mate showed higher adherence rates and overall satisfaction than a conventional pen-and-paper food diary [21]. However, although these study adherence indices capture between-person differences, they do not necessarily reflect the within-person adherence rate during the study, which is essential for a valid mobile image-based assessment of dietary intake (see also [22-24]). Therefore, the aim of this study was to assess both between- and within-person adherence rates for mobile image-based dietary assessments (MIDAs).

Research in the field of Ecological Momentary Assessment (EMA) [24-27] suggests that mobile dietary assessments can be classified as *event-based* monitoring, in which assessments are triggered by the occurrence of a predefined event of interest [28]. Typically, the participants themselves determine when the event has occurred and initiate an assessment [29,30], although some approaches are also developing methods to automatically detect an eating event using mobile sensing (eg, eButton [31] or AIM (automatic ingestion monitor) [32]). As the number of eating events can differ both between and within participants over time, assessing whether events have occurred that were not recorded (*missing events*) is a challenge.

Schembre et al [24] recently reviewed 20 mobile diet EMA studies and found that none of the 10 event-based studies that were included provided data related to adherence to dietary data collection protocols or the number of eating events captured (2 studies reported intake events but summarized eating and drinking occasions [33,34]). In addition, Maugeri and Barchitta [35] reviewed 54 articles and concluded that most mobile diet EMA studies did not report user response or compliance rates. Operationalizing missing event rates is particularly cumbersome because adhering to an event-based dietary collection protocol requires all eating events to be actively reported by the participants (*momentary adherence*).

We suggest a synopsis of 3 different indicators to approximate the rate of missing events in MIDAs. First, one criterion for roughly estimating the number of missing events is to compare the number of eating events reported with the number of events expected based on social norms and observational data. In Western countries, a pattern of 3 meals per day (ie, breakfast, lunch, and dinner) has become normative since the 19th century (Grignon and Grignon, 2004 [36] in French cited by Lhuissier [37]), which suggests that participants should record at least 3 eating events per day. However, observational data derived from 24-hour dietary recalls suggest at least 4 eating episodes per day. Specifically, in the United States, an average of 4.3 eating occasions was recorded per day, of which 3 were main meals [38-40]. Similarly, in Germany, the World Health Organization MONICA (MONItoring CArdiovascular disease) study showed an average of 3 main meals plus 1 snack per day, using a 7-day food intake protocol [40]. Corroborating these findings, a substudy of the European Prospective Investigation into Cancer and Nutrition (EPIC)-Potsdam cohort observed 4 peaks in food consumption, that is, breakfast, lunch, afternoon snack, and dinner [39]. Similarly, assessing eating behavior in the moment using an Ecological Momentary Assessment revealed an average of 3.65 eating occasions per participant across 2 weeks [18]. Using 3- and 4-meal patterns as social comparison standards allows the overall rate of missing events to be estimated with respect to social norms and observational data. Second, logging trajectories and the stability of recorded eating events over time might also indicate the rate of missing events. Assuming that adherence motivation declines over time, the frequency of missing events should generally increase over time, which is mirrored in a declining overall logging trajectory. Moreover, one could also assume that missing events increase over time depending on the quality of the logged food (eg, decreased reporting of irregular meals, such as snacks), indicating selective reporting over time. Therefore, declining logging trajectories of eating events over time can be examined as an additional indicator for missing events. Third, another criterion could be to assess the number of perceived missing events as part of the user experience. However, to the best of our knowledge, no previous studies have assessed these three different indicators to estimate the rate of missing events.

Moreover, the mobile image-based food recording app itself might include features that have an impact on the missing event rates. They can vary in whether and how often they prompt the participants to record their eating events, which raises the question of whether mobile apps with reminders yield higher eating event logging rates, and thus lower missing event rates, than those without prompts. Similarly, the possibility of adding meals after the actual eating events occurred instead of in the moment of eating (referred to as *addendum*) might also have a positive impact on the rate of missing events. In addition, assessing skipped meals (ie, omission or lack of consumption of a meal) allows for differentiation between skipped meals and missing events, which might increase overall measurement precision.

Three EMA studies were conducted using a mobile image-based app to assess eating events in real life to investigate the occurrence of and perceived reasons for missing events (both meals and snacks) by (1) comparing the reported number of eating events with the number of social normative expected events, (2) calculating logging trajectories over time and in dependence of the type of meal, and (3) assessing the number of and reasons for perceived missing events as part of the user experience.

The measurement periods vary across different disciplines and depend on the goals of the study (eg, dietary assessment or intervention). Different methods (eg, food frequency questionnaire, 24-hour recalls, and weighed food records) are used for dietary assessment, which is the focus of this paper. Although assessment periods vary, they typically do not exceed 8 days [41]. For example, 3- to 5-day nonconsecutive 24-hour recalls or up to 7-day weighed food records are used to assess habitual intake. Sharp et al [16] summarized mobile phone–based dietary intake assessment studies, showing that assessment periods typically range from 1 to 7 days. Accordingly, we chose an 8-day assessment period and followed the recommendation of the Food and Agriculture Organization of the United Nations [41] to ensure that we included weekdays and weekends.

The 3 studies used apps with varying technical intensities, ranging from a *basic* MIDA to a more technically *enriched* app to get insights into the impact of additional technical features on logging rates. Study 1 investigated the number of recorded meals and snacks using a custom-programmed smartphone app in which participants were asked to select the meal type from 5 predefined options (breakfast, lunch, afternoon tea, dinner, and snack), take a picture of the eating event, and add a written description of the meal or snack. To develop a better understanding of the typology of missing events, the participants were invited to a semistructured interview in which they were asked to estimate the number of and reasons for missing events (*perceived missing events*) when the MIDA period was over.

Study 2 extended study 1 by adding reminders and making it possible to record eating events after they occurred instead of in the moment but still during the EMA period (*addenda*). As in study 1, semistructured interviews were conducted after the assessment period to probe the perceived number of and reasons for missing events.

In line with study 2, study 3 offered reminders and the possibility of recording eating events after they occurred (*addenda*). Extending studies 1 and 2, participants could also indicate whether they had skipped a meal or snack throughout the day (*skipped meal*). Moreover, the occurrence of and reasons for missing events were assessed on a daily basis during the mobile food record assessment period instead of at the end of the recording period.

In the 3 EMA studies, we examined the following hypotheses:

1. In accordance with the suggested synopsis of 3 different indicators to approximate the rate of missing events in mobile dietary assessments, we compared the number of logged eating events with the number of expected meals based on social norms and observational data, and the number of perceived missing events to test for an overall underreporting of eating events and an overall estimate of missing rate events. In addition, we examined overall and meal type–specific logging trajectories over time to test the hypothesis that adherence motivation decreases and missing events increase over time.
2. We determined whether the number of missing events can be lowered by additional technical features, such as reminders, and options such as adding meals after the actual eating events occurred (*addenda*) and recording skipped meals.
3. Finally, reported reasons for missing events were analyzed to provide relevant information for designing mobile diet apps.

## Methods

### Participants and Ethics

Participants were generally healthy adults (≥18 years) and volunteers recruited from the student and employee population of the University of Konstanz. All participants were reimbursed for their participation. The ethics committee of the University of Konstanz approved the study protocols for all 3 studies. The participants provided written informed consent before enrollment, and all studies adhered to the guidelines of the German Psychological Society and the Declaration of Helsinki.

### Procedure

The participants were recruited via leaflets distributed at the University of Konstanz and postings on Facebook groups. Participants in studies 2 and 3 were also recruited through the web-based recruitment platform for research studies at the University of Konstanz (SONA). Only registered users can sign up for studies via the platform.

The participants were invited to the laboratory for an introductory and closing session either individually (for studies 1 and 3) or in groups (for study 2). At the introductory session, after the participants had completed a questionnaire that assessed demographic variables and their dietary style, they were provided and familiarized with the MIDA.

#### Mobile Image-Based Dietary Assessment

The participants were asked to record all eating events, whether main meals or snacks, for 8 consecutive days. They were specifically asked to indicate the type of meal with the following 5 options: breakfast, lunch, afternoon tea, dinner, and snack. In Germany, *afternoon tea* is called *Kaffee und Kuchen*, which directly translates as *coffee and cake*. It is similar to the idea of a traditional *afternoon tea* meal in the United Kingdom. Specifically, in Germany, people have *Kaffee und Kuchen* in the afternoon (between 4 and 5 PM), typically serving coffee (or tea) with some cake or cookies. Afterward, participants were asked to take a picture of each eating event and provide a short description of the meal or snack (eg, pasta with tomato sauce or oats, milk, apple). Additional courses and leftovers were also recorded by taking pictures. A valid recorded eating event had to include all 3 aspects: (1) meal type, (2) eating event picture, and (3) description of the food. Incomplete entries, missing one or more aspects, were not coded as an eating event. Participants were asked to start recording their meals the day after the introductory session, which usually took place on Monday, Tuesday, or Wednesday. Starting days were limited to the beginning of the week to ensure that any potential issues raised by the participants when they started using the app could be addressed before the weekend began, thus ensuring that data collection would take place both during the week and on weekends.

#### Assessment of Perceived Missing Events: Semistructured Interviews and Open Questions

In studies 1 and 2, the participants were invited back to the laboratory after 8 days of recording for a semistructured interview, which was conducted by 4 trained interviewers (KZ, LK, KV, and DW) to assess the occurrences of and reasons for perceived missing events. All interviews were voice recorded. The participants were asked in an open-question format whether they remembered any occasions during the 8 days of the study period when they did not record a main meal (breakfast, lunch, or dinner) or a snack. If they reported that they had failed to record a main meal or a snack (*perceived missing event*), they were asked to state how often this had happened and why. In study 1, they were also asked about any situational constraints that might have prevented them from recording a particular meal or snack. In study 3, the reasons for missing events were assessed in an open-question format via a web-based questionnaire that could be accessed at any time by pressing a button on the app’s home screen. The participants were also reminded of this option at the end of each EMA day. At the beginning of the study, participants could decide for themselves the time in the evening when the reminder would be sent.

### Data Analysis

Statistical analyses for the EMA data were conducted with IBM SPSS (Version 25) and R 3.2.3, using the packages lme4 1.1-11 [42] and lmerTest 2.0-30 [43]. Data were analyzed separately for each of the 3 studies following the same procedure by examining the three different indicators to quantify the rate of missing events in mobile dietary assessments (normative, time stability, and recalled missing events).

First, the number of reported meals was compared with the normative number of expected meals using one-sample *t* tests. The values 24 (for 3 daily meals including breakfast, lunch, and dinner) and 32 (for 4 daily meals, additionally including a snack) were used as normative criteria. The number of logged meals was compared between meal types using repeated-measures analyses of variance (ANOVAs) and followed up by dependent sample *t* tests, for which alpha was lowered to .008 to account for multiple comparisons. Intraindividual differences in logging rates were examined using intraclass correlations (ICCs), which estimate the proportion of variance explained by the participants compared with the total variance.

Second, assuming that adherence motivation declines over time, changes in the number of reported meals over time were analyzed. Multilevel linear modeling [44] was used to account for the data’s hierarchical structure. Logged eating events per day (lower level/level 1) were nested within participants (higher level/level 2). The number of logged eating events was modeled as a function of time within participants to test whether the number of logged eating events changed over time. Separate models were computed for the total number of eating events and for each meal type. First, a random slopes model allowing both intercept and slope to vary was computed to model whether the participants differed both in the mean number of logged eating events and in the relationship between the number of reported events and time. Second, a random intercept model that allowed only the intercept to vary was computed to model whether the participants differed only in the mean number of logged eating events and not in the relationship between the number of logged events in time. If significant, both models were compared using a deviance test [44]. A nonsignificant deviance test indicates that the less complex model (ie, random intercept model) is preferred, whereas a significant deviance test indicates that the more complex model (ie, random slopes model) is preferred. Pseudo-R² was computed as recommended by Raudenbush and Bryk [45].

Third, the number of and reasons for perceived missing events assessed through semistructured interviews (studies 1 and 2) and open questions (study 3) were coded by 2 authors (KZ and LK) and a third independent researcher using a standardized manual developed by KZ, LK, BR, and CB, with input from all authors. The number of missing events for meals and snacks was compared using dependent samples *t* tests.

### Study 1 (8 Days of Mobile Food Recording): Basic Mobile Image-Based Dietary Assessment

The app used for the mobile image-based dietary assessment was programmed using the Android movisensXS app (version 0.8.4203, movisens GmbH Karlsruhe, Germany). The participants could either use their own smartphone or were provided with a study smartphone (ASUS Padfone Infinity, Android 5.0.2, n=10).

### Study 2 (8 Days of Mobile Food Recording): The Effect of Reminders and Addenda

Other than a few amendments, the procedure was identical to that of study 1. The participants were provided with a study smartphone (ASUS Padfone Infinity, Android 5.0.2), and the MIDA was realized with the SMARTFOOD app, which was developed as part of the research project SMARTACT [2,18,46-48] and included a feature to set a reminder in the morning to record food intake. The participants were asked to set the reminder at the beginning of the study during the introduction session. The app also had an addendum feature to log eating events that participants missed to record in the moment of consumption, which was enabled on half of the devices (n=18). Anthropometric measures were assessed during the introductory session, and the participants received a booklet explaining how to use the smartphone and app for recording food intake.

### Study 3 (8 Days of Mobile Food Recording): Reporting Missing and Skipped Events During the Assessment Period

The procedure was identical to those of study 1 and 2, with the following amendments. The optional study smartphone (n=58) was either an ASUS Padfone Infinity (Android 5.0.2) or a Samsung Galaxy J5 (Android 6.0.1), with a custom-programmed mobile app (version 0.8.4203, movisens GmbH Karlsruhe; Germany) preinstalled.

In the app, participants could indicate whether they had skipped a meal or snack, and they could record eating events later (*addenda*), including meal type, composition, and the reasons for missing logging it. Moreover, the participants were asked to set customized reminders in the morning and evening. Extending study 2, an evening reminder was sent to remind the participants to (1) record any missing event and the respective reasons, and (2) log any skipped meals or snacks.

## Results

### Study 1 (8 Days of Mobile Food Recording): Basic Mobile Image-Based Dietary Assessment

The aim of study 1 was to examine the number of and reasons for missing events during event-based mobile food recording in real life for 8 consecutive days according to 3 different indicators (normative, time stability, and recalled missing events). The study investigated the number of recorded meals and snacks using a custom-programmed smartphone app. Participants logged the meal type, captured an image of the eating event, and added a written description of the meal or snack (*eating event*). The app used a *basic* MIDA as it did not include reminders or the possibility of adding eating events after they had occurred (*addenda*) or recorded skipped meals.

The 38 participants (28/38 female, 74%; 33/38 students, 87%) who took part in the study had a mean age of 24.5 years (SD 5.88, range 18-48). Of the 38 participants, 21 (55%) were omnivores, 7 (18%) vegetarians, 3 (8%) vegans, and 7 (18%) adhered to other dietary styles. As compensation, they either received course credits (2.0 h; n=10) or took part in a lottery to win one of 4 €25 (US $30; n=28) Amazon vouchers.

None of the participants dropped out of the study, indicating an excellent overall retention. The participants logged a total of 1099 eating events over the 8-day study period. Of these, 29 entries (2.64%) were canceled by the participant before completing the food recording, resulting in a total of 1070 recorded eating events (Table 1). Control analysis showed that participants who used their own vs a loaned smartphone did not differ significantly with respect to the number of reported meals (*t*s_36_≤|1.46|, *P*s≥.15, *d*s≤0.49) or the number of reported perceived missing events (main meals: t_32_=−1.11, *P*=.28, *d*=0.40; snacks: t_29_=−0.52, *P*=.61, *d*=0.22).

**Table 1:** Logged and social normative expected eating events by self-classified meal type over 8 days for studies 1, 2, and 3.

Meal type			Absolute number of meals (%)		Mean (SD)		Min/max		Difference observed-normative meals^a^		*t* value		*P* value		Cohen *d*		ICC^b^
**Study 1 (N=38, *df*=37)**																	
	Breakfast	244 (22.8)		6.42 (2.04)		2/11		−1.58		–4.78		<.001		0.78		.13	
	Lunch	213 (19.9)		5.61 (1.59)		1/9		−2.40		−9.31		<.001		1.51		.04	
	Dinner	256 (23.9)		6.74 (2.20)		0/11		−1.26		−3.54		.001		0.57		.09	
	Snacks	330 (30.8)		8.68 (6.97)		0/27		0.68		0.61		.55		0.10		.10	
	Afternoon tea	27 (2.5)		0.71 (1.18)		0/4		—^c^		—		—		—		.38	
	Total	1070 (100)		28.16 (9.57)		12/47		−3.84		−2.48		.02		0.40		.38	
**Study 2 (N=35, *df*=34)**																	
	Breakfast	241 (25.8)		6.89 (2.37)		0/13		−1.11		−2.78		.01		0.47		.35	
	Lunch	211 (22.6)		6.03 (1.77)		1/9		−1.97		−6.58		<.001		1.11		.07	
	Dinner	234 (25.1)		6.69 (1.92)		1/10		−1.31		−4.05		<.001		0.68		.10	
	Snacks	222 (23.8)		6.34 (4.81)		0/17		−1.66		−2.04		.049		0.34		.30	
	Afternoon tea	26 (2.8)		0.74 (1.25)		0/6		—		—		—		—		.18	
	Total	934 (100)		26.69 (7.46)		11/42		−5.31		−4.21		<.001		0.71		.30	
**Study 3 (N=110, *df*=109)**																	
	Breakfast	828 (23.9)		7.53 (2.58)		0/23		−0.47		−1.92		.06		0.18		.26	
	Lunch	744 (21.4)		6.76 (1.78)		1/10		−1.24		−7.30		<.001		0.70		.06	
	Dinner	832 (24.0)		7.56 (1.96)		1/13		−0.44		−2.34		.02		0.22		.06	
	Snacks	953 (27.5)		8.66 (6.23)		0/33		0.66		1.12		.27		0.11		.35	
	Afternoon tea	112 (3.2)		1.02 (1.11)		0/5		—		—		—		—		.02	
	Total	3469 (100)		31.54 (8.73)		10/61		−0.46		−0.56		.58		0.05		.32	

^a^Reference *t* value was set to a value of 8 meals for individual meal types and to a value of 32 meals for total meals; negative values indicate fewer observed than normative expected number of meals.

^b^ICC: intraclass correlation.

^c^For afternoon tea no normative value was set and therefore no difference between observed and normative meals was calculated.

#### Logged Versus Social Normative Expected Number of Meals

On the group level and across eating events, the participants logged an average of 28.16 (SD 9.57) meals and snacks during the 8 study days. Thus, at the group level, the number of recorded eating events concurred with the social normative expected number of meals, which ranged between 24 and 32 (24 for 3 daily meals including breakfast, lunch, and dinner and 32 for 4 daily meals including an additional snack). A considerable variability in the number of entries emerged at the person level, ranging from 12 to 47 entries. However, the majority (23/38, 61%) logged 24 or more eating events during the study period. Testing further intraindividual differences in the overall logging rate yielded an ICC of ρ=0.38 across eating events, indicating that 62% of the overall variation in logged occasions was because of variation within participants, rather than variations in the logging rates between individuals.

As Table 1 shows, across the 8 days, the participants logged 244 breakfasts, 213 lunches, 27 afternoon teas, 256 dinners, and 330 snacks. On average, they recorded 6.42 (SD 2.04) breakfasts, 5.16 (SD 1.59) lunches, 6.74 (SD 2.20) dinners, 8.68 (SD 6.97) snacks, and 0.71 (SD 1.18) afternoon teas. Comparing the average number of recorded main meals (breakfast, lunch, and dinner) and snacks with the social normative expected number shows that the number of main meals and snacks recorded over the study period was significantly lower than the normative expected figure of 8, t_37_=−2.48, *P*=.02, *d*=0.40. Moreover, a repeated-measures ANOVA (Greenhouse-Geisser corrected, ε=.45) with the factor *meal type* (breakfast, lunch, dinner, and snack) yielded a significant main effect with *F*_1.35,49.76_=5.08, *P*=.02, partial η^2^=.12, indicating that the number of logged meals varied depending on the meal type. Subsequent *t* tests indicated that significantly fewer lunches than dinners (t_37_=−3.30, *P*=.002, *d*=0.59) and snacks (t_37_=−2.90, *P*=.006, *d*=0.61) were recorded. No other comparisons were statistically significant, with *t*s<|2.17|, *P*s≥.04, exceeding the predetermined α=.008 to correct for multiple comparisons. Examining intraindividual differences in logging rates for the different main meals and snacks yielded ICC coefficients of ρ≤0.38, suggesting that the nesting of logged events within individuals was not substantial. Hence, overall, interindividual differences in logging rates were very small.

#### Logging Trajectories of Eating Events Over Time

**Figure 1:**
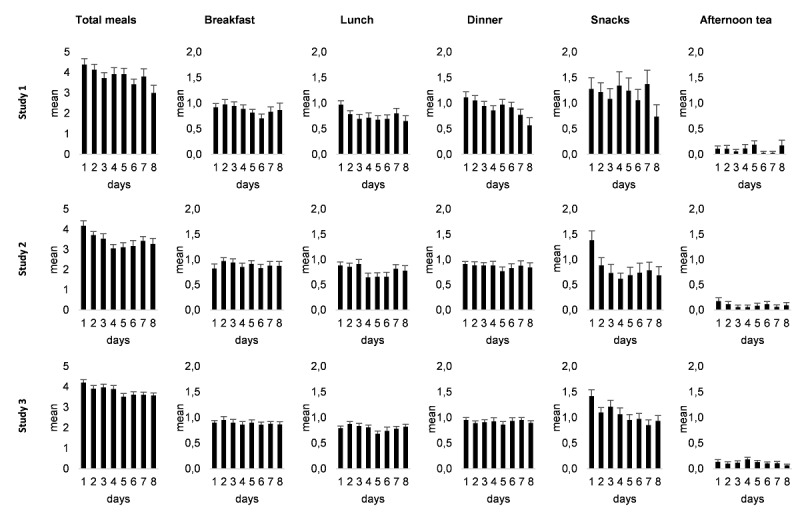
Average logging trajectories across the 8-day study period for studies 1, 2 and 3 across all meals and by meal types. Bars represent mean logged meals; error bars represent the standard error of the mean.

Logging trajectories over time were tested using multilevel modeling. Specifically, models were computed to test whether there was a significant linear change in the total number of logged eating events across the 8-day study period (Figure 1 and Table 2).

**Table 2:** Multilevel models for logging trajectories across the 8-day study period across all eating events and by meal types for studies 1, 2, and 3.

Characteristics			Random slopes model (fixed effects)					Random intercept model (fixed effects)				
			b	SE	*t*	*df*	*P* value	b	SE	*t*	*df*	*P* value
**Study 1 (N=38)**												
	**Model 1: Across meal types**											
		Intercept	4.39	0.24	18.01	34.73	<.001	4.39	0.26	16.76	98.74	<.001
		Days	−0.14	0.04	−3.09	35.26	.004	−0.14	0.04	−3.52	235.91	<.001
	**Model 2: Breakfast**											
		Intercept	0.97	0.07	13.22	35.73	<.001	0.97	0.07	13.85	180.70	<.001
		Days	−0.02	0.02	−1.28	36.14	.21	−0.02	0.01	−1.65	237.44	.10
	**Model 3: Lunch**											
		Intercept	0.86	0.07	11.60	36.12	<.001	0.87	0.07	12.66	232.83	<.001
		Days	−0.03	0.02	−1.59	36.22	.12	−0.03	0.01	−1.99	240.80	.048
	**Model 4: Afternoon tea**											
		Intercept	0.11	0.05	2.03	41.17	.049	0.11	0.05	2.20	193.69	.023
		Days	−0.00	0.01	−0.19	102.55	.85	−0.00	0.01	−0.17	235.84	.86
	**Model 5: Dinner**											
		Intercept	1.15	0.08	13.93	78.52	<.001	1.15	0.08	13.77	204.69	<.001
		Days	−0.06	0.02	−3.49	230.13	<.001	−0.06	0.02	−3.50	239.04	<.001
	**Model 6: Snacks**											
		Intercept	1.31	0.16	8.02	64.39	<.001	1.30	0.20	6.45	99.12	<.001
		Days	−0.04	0.03	−1.08	81.43	.28	−0.03	0.03	−1.05	235.58	.30
**Study 2 (N=35)**												
	**Model 1: Across meal types**											
		Intercept	3.94	0.18	21.60	33.33	<.001	3.93	0.20	19.73	100.64	<.001
		Days	−0.12	0.03	−3.80	34.10	<.001	−0.12	0.03	−3.86	236.57	<.001
	**Model 2: Breakfast**											
		Intercept	0.91	0.07	12.29	34.56	<.001	0.91	0.07	13.90	97.40	<.001
		Days	−0.01	0.01	−0.43	34.77	.67	−0.01	0.01	−0.53	237.20	.60
	**Model 3: Lunch**											
		Intercept	0.87	0.06	13.92	225.78	<.001	0.87	0.07	13.00	213.60	<.001
		Days	0.02	0.01	−1.60	67.86	.12	−0.02	0.01	−1.65	238.35	.10
	**Model 4: Afternoon tea**											
		Intercept	0.13	0.05	2.46	34.35	.02	0.13	0.04	3.14	155.22	.002
		Days	−0.01	0.01	−0.89	33.93	.38	−0.01	0.01	−1.10	237.97	.27
	**Model 5: Dinner**											
		Intercept	0.91	0.06	15.26	100.36	<.001	0.91	0.06	14.56	187.11	<.001
		Days	−0.01	0.01	−1.00	166.84	.32	−0.01	0.01	−0.98	234.57	.33
	**Model 6: Snacks**											
		Intercept	1.12	0.14	7.88	40.09	<.001	1.12	0.14	7.95	104.59	<.001
		Days	−0.07	0.02	−3.23	236.17	.001	−0.07	0.02	−3.23	236.87	.001
**Study 3 (N=110)**												
	**Model 1: Across meal types**											
		Intercept	4.49	0.14	32.81	125.39	<.001	4.49	0.13	34.57	313.44	<.001
		Days	−0.11	0.02	−5.47	635.68	<.001	−0.11	0.02	−5.52	753.99	<.001
	**Model 2: Breakfast**											
		Intercept	1.01	0.04	23.89	145.02	<.001	1.01	0.04	24.10	382.80	<.001
		Days	−0.01	0.01	−1.84	751.63	.07	−0.01	0.01	−1.84	754.77	.07
	**Model 3: Lunch**											
		Intercept	0.89	0.04	25.30	737.26	<.001	0.9	0.04	24.30	710.03	<.001
		Days	−0.01	0.01	−0.86	378.69	.39	−0.01	0.01	−0.89	755.33	.38
	**Model 4: Afternoon tea**											
		Intercept	0.16	0.03	5.42	131.39	<.001	0.16	0.03	5.86	770.79	<.001
		Days	−0.01	0.01	−1.33	251.58	.18	−0.01	0.01	−1.36	753.21	.17
	**Model 5: Dinner**											
		Intercept	0.98	0.04	24.43	344.77	<.001	0.98	0.04	24.24	700.66	<.001
		Days	−0.00	0.01	−0.45	740.08	.65	−0.00	0.01	−0.45	753.67	.65
	**Model 6: Snacks**											
		Intercept	1.44	0.11	13.66	119.67	<.001	1.45	0.10	14.18	290.82	<.001
		Days	−0.08	0.01	−5.32	523.12	<.001	−0.08	0.01	−5.41	753.06	<.001

Overall, there was a small but statistically significant negative trend over time (b=−.14, t_235.91_=−3.52, *P*<.001, pseudo-R²=0.05), indicating that the number of logged eating events decreased over time. However, as the participants did not vary with respect to the time trend as the random intercept model was preferred (*χ*²_2_=3.5, *P*=.18), the trend was generalizable across participants.

For individual meal types, small and significant negative time trends emerged for lunches (b=−.03, t_240.80_=−1.99, *P*=.048, pseudo-R²=0.01) and dinners (b=−.06, t_239.04_=−3.50, *P*<.001, pseudo-R²=0.04). Accordingly, the number of logged lunches and dinners decreased across the 8 consecutive days. Again, the random intercept model was preferred (*χ*²_2_≤3.7, *P*s≥.16), indicating that the observed time trends were comparable between participants. There was no significant change in logging frequency over time for other meal types, including snacks, breakfasts, and afternoon teas (Table 2).

#### Number of Reasons for Perceived Missing Events

In total, 35 of the 38 participants (92%) reported in the interview that they had missed logging at least one eating event during the study period, with a range from 1 to 14 (median 2.50, mean 4.47, SD 3.95; 5 participants could not specify the frequency of missing events). Moreover, 30 of these 35 participants (86%; 4 did not specify the frequency) stated that they had missed reporting at least one snack, and 22 of 35 (63%; one did not specify the frequency) had missed at least one main meal. Perceived missing event rates ranged between 1 and 9 for main meals (median 1.00, mean 1.73, SD 2.26) and between 1 and 8 for snacks (median 1.5, mean 2.73, SD 2.63), t_29_=−1.89, *P*=.07, *d*=0.41.

In total, 69 different reasons were provided by the 35 participants who reported at least one missing event. Of these, 12 participants specified 1 reason, 17 specified 2, and 6 gave between 3 and 5 reasons (median 1.97, SD 1.0).

Of these 69 reasons, 60 provided information that went beyond merely stating that an eating event was missed, and these were categorized into 6 different categories (Table 3). The most frequently mentioned reason for missing an event was multitasking in the moment of eating (eg, “I forgot to record it because I was at a party.”), which covered 37 of the 69 reasons (54%) provided. In addition, when a participant only gave a single reason for not reporting an eating event, the most common reason was multitasking (5/12 single reasons, 42%). As Table 3 shows, the types of multitasking can be further divided into occasions when participants were unaware that they had missed recording a meal (eg, because they were too busy: 33/69, 48%), and occasions when participants had deliberately decided against recording because of situational barriers (eg, time pressure: 4/69, 6%).

**Table 3:** Type and number of reported reasons for perceived missing events for studies 1, 2, and 3.

Characteristics		All reasons		Single reasons^a^		Examples
Type of reason		Total, n (%)	Main^b^/snacks, n	Total, n (%)	Main^b^/snacks, n	
**Study 1 (n=35)^c^**						
	Multitasking	37 (53.6)	10/27	5 (41.7)	2/3	N/A^d^
	Not being aware	33 (47.8)	10/23	4 (33.3)	2/2	“I forgot to record because I was at a party.”
	Deliberately deciding against	4 (5.8)	0/4	1 (8.3)	0/1	“It was too awkward to record the second helping.”
	Device-related obstacles	13 (18.8)	7/6	2 (16.7)	0/2	N/A
	Device malfunction	3 (4.3)	2/1	0	0	“The phone ran out of battery.”
	No device	10 (14.5)	5/5	2 (16.7)	0/2	“I left my smartphone at home.”
	Situational barriers	10 (14.5)	2/8	2 (16.7)	0/2	N/A
	Practical reasons	9 (13.0)	1/8	2 (16.7)	0/2	“I was at work.”
	Social reasons	1 (1.4)	1/0	0	0	“I was eating with other people.”
	Not further specified	9 (13.0)	6/3	3 (25.0)	3/0	“I forgot.”
	Total	69 (100)	25/44	12 (100)	5/7	N/A
**Study 2 (n=28)^e^**						
	Multitasking	15 (34.1)	11/4	8 (53.3)	8/0	N/A
	Not being aware	11 (25.0)	10/1	7 (46.7)	7/0	“I was deep in a conversation and did not think about recording.”
	Deliberately deciding against	4 (9.1)	1/3	1 (6.7)	1/0	“It was too awkward to record the chips.”
	Device-related obstacles	14 (31.8)	11/3	3 (20.0)	2/1	N/A
	Device malfunction	1 (2.3)	1/0	0	0	N/A
	No device	13 (29.5)	10/3	3 (20.0)	2/1	“I knew that I would go partying after eating at a friend’s house, and I didn’t want to take the smartphone to the club.”
	Situational barriers	11 (25.0)	5/6	3 (20.0)	1/2	N/A
	Practical reasons	6 (13.6)	3/3	2 (13.3)	1/1	“I am not allowed to use my smartphone at work.”
	Social reasons	5 (11.4)	2/3	1 (6.7)	0/1	“I felt awkward when recording my snack in front of other students.”
	Not further specified	4 (9.1)	2/2	1 (6.7)	1/0	“I forgot to take a picture.”
	Total	44 (100)	29/15	15 (100)	12/3	N/A
**Study 3 (n=99)^f^**						
	Multitasking	70 (19.4)	49/21	5 (25.0)	4/1	N/A
	Not being aware	39 (10.8)	25/14	3 (15.0)	3/0	“I only remembered after my lunch box was empty.”
	Deliberately deciding against	31 (8.6)	24/7	2 (10.0)	1/1	“I did not have the time.”
	Device-related obstacles	98 (27.1)	68/30	11 (55.0)	10/1	N/A
	Device malfunction	22 (6.1)	17/5	2 (10.0)	2/0	“The app did not work properly.”
	No device	76 (21.1)	51/25	9 (45.0)	8/1	“I left my smartphone at home.”
	Situational barriers	48 (13.3)	20/28	0	0	N/A
	Practical reasons	35 (9.7)	12/23	0	0	“I could not use my phone during the lecture.”
	Social reasons	13 (3.6)	8/5	0	0	“I was on a date.”
	Not further specified	145 (40.2)	86/59	4 (20.0)	3/1	“I forgot to take a picture.”
	Total	361 (100)	223/138	20 (100)	17/3	N/A

^a^Only 1 reason was provided by the participant.

^b^Main meals (breakfast, lunch, and dinner).

^c^In study 1, 35 of 38 participants reported a missing event.

^d^N/A: not applicable.

^e^In study 2, 28 out of 35 participants reported a missing event.

^f^In study 3, 99 out of 110 participants reported a missing event.

Device-related obstacles were the second most frequent reason (13/69 reasons, 19%). These obstacles can be further divided into those related to device malfunctions such as the app not working or the battery was low (3/69 reasons, 4%), and occasions when the participants did not have the device with them (eg, it was not at hand; 10/69 reasons, 15%). Finally, situational barriers that prevented the participants using the mobile device either because it was not sufficiently feasible or admissible accounted for 10 of the 69 reasons (15%). These situational barriers could be further divided into practical reasons such as “going somewhere,” “driving,” or “not having a hand free,” with 9 of the 69 reasons (13%), whereas social reasons such as feeling intimidated by taking a picture were only noted once (1/69, 2%).

### Study 2 (8 Days of Mobile Food Recording): The Effect of Reminders and Addenda

Mobile apps currently vary as to whether and how often they prompt the participants to record their eating events, which raises the question of whether mobile apps with reminders yield higher eating event logging rates than those without prompts. Similarly, the possibility of adding meals after the actual eating events has occurred (*addenda*) might also have a positive impact on the rate of missing events. Therefore, study 2 extended study 1 by implementing reminders and the possibility to record missing eating events after they had occurred but still during the food assessment period (*addenda*).

A total of 35 participants (31/35 female, 89%; 32/35 students, 91%) took part in the study, with a mean age of 25.5 years (SD 5.69, range 19-41) and (objectively measured) BMI of 22.5 kg/m^2^ (SD 5.5, range 15-43). Of the 35 participants, 20 (57%) were omnivores, 5 (14%) vegetarians, 4 (11%) vegans, and 6 (17%) adhered to other dietary styles. As compensation, the participants received either course credits (3.0 h; n=5) or €25 (US $30; n=30).

None of the participants dropped out of the study, which again indicates excellent retention. A total of 1093 eating events were logged by the participants over the 8-day time period. Of these, 159 entries (14.55%) were canceled before completion, resulting in a total of 934 complete eating event records, of which 888 were recorded during the eating event and 46 (4.9%) were recorded belatedly (*addenda*).

#### Logged Versus Social Normative Expected Number of Meals

As Table 1 shows, on average, the participants logged 26.69 (SD 7.46) eating events across the 8-day study period. Overall, at the group level, the number of logged eating events was within the social normative expected range of 24 to 32 eating events but significantly below the 32 meals threshold, t_34_=−4.21, *P*<.001, *d*=0.71. As in study 1, a substantial variability in the number of reported eating events occurred at the person level, ranging from 11 to 42 entries per participant. The majority (22/35, 63%) logged 24 or more eating events across the 8 days, and the ICC of ρ=0.30 indicates that 70% of the observed variance in the number of logged eating events was due to differences within, rather than between, participants.

The observed number of logged breakfasts, lunches, dinners, and snacks ranged between 6.03 (SD 4.81, lunch) and 6.89 (SD 2.37, breakfast) and were significantly lower than the social normative expected 8 daily meals, respectively. The number of logged events did not vary depending on the meal type, as indicated by a repeated measures ANOVA with the factor *meal type* (breakfast, lunch, dinner, snack), *F*_1.78,60.59_=0.69, *P*=.49, partial η²=.02 (Greenhouse-Geisser corrected, ε=.59). The total number of logged meals and the number of logged meals per meal type did not differ between those participants who had access to the addendum feature (n=18) and those who did not (n=17), *t*s_33_≤|0.85|, *P*s≥.40, *d*s≤0.29 (Multimedia Appendix 1). Examining intraindividual differences in logging rates for the different meal types yielded small ICC coefficients (ρ≤0.35), suggesting that the nesting of logged events within individuals was not substantial. Hence, overall, interindividual differences in logging rates were small.

#### Logging Trajectories of Eating Events Over Time

Figure 1 depicts the logging trajectories for the 8-day study period. Again, logging trajectories over time were analyzed using multilevel modeling (Table 2 and Figure 1).

Overall, there was a small but statistically significant negative trend over time (b=−.12, t_236.57_=−3.86, *P*<.001, pseudo-R²=0.06), indicating that fewer eating events were reported as the study progressed. The random intercept model was preferred (*χ*²_2_=1.1, *P*=.57), which indicates that the trend was generalizable across the participants.

Although there were no significant changes in logging frequencies over time for the 4 main meal types (Table 2), a small and significant negative trend emerged for snacks (b=−.07, t_236.87_=−3.23, *P*=.001, pseudo-R²=0.04), indicating that the number of logged snacks decreased significantly across the 8 days. The random intercept model was again preferred (*χ*²_2_=0.0, *P*>.99), indicating that the trends were comparable between the participants.

A visual inspection of Figure 1 suggests that the number of logged snacks dropped between days 1 and 2. Examining daily logging frequencies for snacks (Multimedia Appendix 1) showed that there was also a shift toward reporting fewer *multiple* snacks between day 1 and day 2. Specifically, the number of participants who recorded 2 to 3 snacks decreased from 16 to 8, whereas those who reported 1 snack increased from 9 to 13. Similar but less pronounced changes occurred between days 2 and 3, and between days 3 and 4.

#### Number of Reasons for Perceived Missing Events

In the interviews that followed the EMA assessment period, 28 of the 35 participants (80%) reported that they had missed recording at least one eating event (range 1-9 events, median 2.00, mean 2.12, SD 1.88; 5 participants could not state the frequency of missing events). Furthermore, 24 of these 28 participants (86%; 3 did not specify the frequency) stated that they had missed at least one main meal, and 11 of the 28 participants (39%) that they had missed at least one snack. Perceived missing events were significantly higher for main meals (median 1.00, mean 1.48, SD 1.16, range 1-5) than for snacks (median 0.00, mean 0.74, SD 1.39, range 1-6, t_22_=2.10, *P*=.047, *d*=0.58).

In total, 44 different reasons were provided by the 28 participants who reported a missing event, of which 15 participants specified 1 reason, 10 gave 2 reasons, and 3 participants provided 2 reasons (mean 1.57, SD 0.69).

As Table 3 shows, multitasking in the moment of eating was again the most frequently mentioned reason for missing an event (15/44 reasons, 34%), whereas 11 of 44 (25%) listed not being aware of and 4 of 44 (9%) deliberately deciding against recording an event. The second most common reason was device-related obstacles (14/44, 32%), of which device malfunctions (1/44, 2%) was less frequent than having no device (13/44, 30%). Situational barriers were mentioned 11 of 44 times (25%), of which practical reasons (6/44, 15%) were slightly more frequent than social reasons (5/44, 11%).

### Study 3 (8 Days of Mobile Food Recording): Reporting Missing and Skipped Events During the Assessment Period

The *enriched* MIDA in study 2 yielded a similar overall pattern of results to study 1, demonstrating that including a fixed reminder in the morning did not increase the number of logged events. However, one might argue that a fixed daily reminder set at the beginning of the study might not be effective because individual time schedules might vary between and within participants. Thus, in study 3, we tested whether a fixed reminder in the evening, in addition to a daily reminder in the morning, would decrease the rate of missing events. Moreover, to distinguish missing events from skipped eating events, we also included the possibility for participants to indicate the omission or lack of consumption of a meal or snack. Further extending studies 1 and 2, the participants could indicate the occurrence of and reasons for missing events on a daily basis during the MIDA period to reduce potential memory effects.

A total of 113 participants were recruited. Among them, 2 dropped out during the 8-day recording and 1 recorded only 1 eating event, resulting in a final sample of 110 participants (91/110 female, 82.7%; 106/110 students, 96.4%) with a mean age of 22.02 years (SD 5.30, range 18-51) and (self-reported) BMI of 21.9 kg/m^2^ (SD 3.44, range 17-44). Of the 110 participants, 66 (60.0%) were omnivores, 18 (16.4%) vegetarians, 8 (7.3%) vegans, and 18 (16.4%) adhered to other dietary styles. The participants received course credits (1.5 h; n=70) or €15 (US $18; n=40) as compensation for their participation.

As of the 113 participants, only 2 dropped out of the study and 1 recorded only 1 meal, a very good retention rate (97.4%) is indicated. In total, the participants logged 3365 eating events over the 8-day study period. Of these, 133 entries (3.95%) were canceled by the participants or were incomplete (eg, picture missing), resulting in a total of 3232 complete eating event records (Multimedia Appendix 1), of which 2871 were recorded at the eating event and 361 (11.17%) were belated recordings (*addenda*). In addition, 86 of 110 participants (78.2%) reported having skipped 245 eating events. No meal type was specified for 8 skipped eating events, resulting in a total of 237 skipped eating events that were added to the eating event records. Hence, the total final sample included 3469 eating events (Table 1). The control analysis showed no significant difference between participants who used their own vs a loaned smartphone with respect to the number of reported meals (*t*s_108_≤|1.81|, *P*s≥.07, *d*s≤0.35) or the number of reported missing snacks (t_97_=1.37, *P*=.17, *d*=0.28). However, they differed in terms of the number of reported missing main meals (t_97_=2.13, *P*=.04, *d*=0.43; mean_own_ 2.62, SD_own_ 2.07, mean_loaned_ 1.86, SD_loaned_ 1.54).

#### Logged Versus Social Normative Expected Number of Meals

On average, the participants recorded 31.54 (SD 8.73) meals and snacks during the 8 study days (Table 1). In contrast to studies 1 and 2, the number of recorded eating events concurs with the social normative threshold value of 32 occasions (4 daily meals and an additional snack, t_109_=−0.56, *P*=.58, *d*=0.05). The number of entries ranged from 10 to 61 per participant, with most (91/110, 82.7%) logging 24 or more eating events during the study period. Majority (68%) of the overall variation in logged occasions was because of variations within participants rather than variations of logging rates between individuals (ICC of ρ=0.32).

Table 1 shows that the observed number of logged lunches (mean 6.76, SD 1.78) and dinners (mean 7.56, SD 1.96) were significantly lower than the social normative expected total of 8 for the study period, whereas the number of logged breakfasts (mean 7.53, SD 2.58) and snacks (mean 8.66, SD 6.23) reached the social normative expected threshold value of 8 occasions. Accordingly, the number of reports differed between meal types, as indicated by a repeated-measures ANOVA, *F*_1.53,167.08_=5.84, *P*=.007, partial η²=.05 (Greenhouse-Geisser corrected, ε=.51). Significantly fewer lunches were recorded than dinners (t_109_=−4.09, *P*<.001, *d*=0.43) and snacks (t_109_=−3.16, *P*=.002, *d*=0.41). All other comparisons were not statistically significant, with *t*s_109_≤−2.66, *P*s≥.009, *d*s≤0.35 (α adjusted to .008 to account for multiple comparisons). ICC coefficients were all ρ≤0.35, suggesting that, overall, interindividual differences in logging rates were small.

The number of reported meals without including skipped meals was also analyzed to examine the effect of including the recording of skipped meals (Multimedia Appendix 1). A total of 3232 eating events were recorded, with an average of 29.38 (SD 8.54) events logged across 8 days, which is significantly lower than the normative threshold of 32 meals (t_109_=−3.22, *P*=.002, *d*=0.31, ICC of ρ=0.34). However, the majority still logged more than 24 meals throughout the study period (82/110, 74.5%). On average, the participants missed 2.62 eating events across the 8 days. The number of missing events also deviated significantly from the normative expected 8 eating events for the different meal types (breakfast mean 6.97, SD 2.64; lunch mean 6.09, SD 1.91; and dinner mean 7.51, SD 2.09; *t*s_109_≥−4.08, *P*s<.001, *d*s≥0.39). However, the observed and normative expected number of logged events concurred for snacks (mean 8.23, SD 5.93; t_109_=.23, *P*=.69, *d*=0.04).

#### Logging the Trajectories of Meals Over Time

Logging trajectories for the 8-day study period were again examined using multilevel modeling (Table 2 and Figure 1). Overall, there was a small but statistically significant negative trend over time (b=−.11, t_753.99_=−5.52, *P*<.001, pseudo-R²=0.04), indicating that fewer meals were reported over time. The random intercept model was preferred (*χ*²_2_=1.3, *P*=.53), which indicates that participants did not vary with respect to time trends.

For individual meal types, small and significant negative trends also emerged for snacks (b=−.08, t_753.06_=−5.41, *P*<.001, pseudo-R²=0.04), indicating that the number of reported snacks decreased across the 8 consecutive days. Again, the random intercept model was preferred (*χ*²_2_=3.1, *P*=.22), demonstrating that the trends were comparable between participants. No significant change in logging frequency over time was observed for the other food types (breakfast, lunch, dinner, and afternoon tea; Table 2).

#### Number of Reasons for Perceived Missing Events

Of the 110 participants, 99 (90.0%) reported that they had missed recording at least one main meal or snack (median 3.00, mean 3.65, SD 2.93, range 1-20). For main meals, 90 of 99 participants (82%) reported at least one missing event, whereas for snacks, missing events were reported by 57 of 99 (52%) participants. On average, the number of missing events for snacks (median 1.00, mean 1.39, SD 1.96) was significantly lower than the number of missing events for main meals (median 2.00, mean 2.25, SD 1.86; t_98_=3.48, *P*=.001, *d*=0.45).

In total, 361 different reasons were provided by the 99 participants who reported having missed logging at least one eating event, of which 20 participants (20%) specified one reason for missing reporting an eating event, 22 (22%) gave 2, and 57 (58%) between 3 and 20 reasons (median 3.00, mean 3.65, SD 2.93).

Of the 361 reasons provided, 216 (59.8%) included information beyond merely stating that an eating event was missed. As Table 3 shows, device-related obstacles (specifically having no device at hand) were the most frequently specified reason for a missing event (98/361, 27.1%). This was also the case when only a single reason was provided (11/20 provided single reasons, 55%). The next most frequent reasons were those related to multitasking (70/361, 19.4%), followed by situational barriers (48/361, 13.3%).

## Discussion

The goal of the 3 studies was to assess the number of and reasons for *missing events* in MIDA in event-based EMA studies, using apps that varied in their technical features. The 3 studies assessed how often eating events (meals and snacks) were missed over a period of 8 days in a naturalistic setting by comparing the number of recorded events (1) with the number of expected events based on social norms and observational data, (2) over time, and (3) with recollections of missing events. We used different apps ranging from a *basic* app (study 1) to a more *enriched* MIDA app, which included individually set reminders to record food intake, and the possibility of recording *addenda* and skipped meals (study 3). To gain a greater insight into the occurrence of missing events, we also assessed the users’ reasons for missing events. To our knowledge, these are the first studies that have assessed individual’s performance by recording dietary food intake across an extended duration in their naturalistic settings.

### Study Attrition

Attrition rates, as an indicator of adherence, are discussed in the specific context of EMA studies because its comparably intensive data collection is presumed to lead to a high perceived burden on the participants and thus to discontinued usage [49]. Moreover, study attrition is essential for estimating momentary adherence or compliance rates [49,50]. All 3 of the studies showed excellent overall study retention, with only 3 of the 189 participants who enrolled dropping out during the 8-day study period (1.6%). Liao et al [23] summarized compliance-related results from 13 EMA studies of diet and physical activity in youth and, although most previous EMA studies reported initial participant enrollment, only 2 studies formally reported attrition rates (ie, the number of participants who dropped out of the study for any reason). Similarly, Schembre et al [24] reviewed the methods used in mobile diet EMA studies without including information on study attrition (see also study by Wen et al [50]). Villinger, Wahl et al [20] recently analyzed 41 app-based intervention studies on nutrition behaviors and nutrition-related health outcomes in adolescents and adults with a total of 6348 participants and found an average attrition rate of 18.7% (SD 16.27, range 0%-72%). This is highly comparable to the average attrition rate of 18% found by Crutzen et al [51] for health behavior change trials. Thus, attrition rates are comparably low even in longitudinal intervention studies, which place a higher burden on the participants than behavior assessment studies. Taken together, these and the findings from this study provide some confidence that using intensive data collection methods including mobile dietary EMA is feasible in terms of participant retention.

### Momentary Adherence and Missing Events Across Studies and Over Time

Overall, 183 participants reported 5473 eating events, including 3803 main meals, 1505 snacks, and 165 afternoon teas. The momentary adherence rate, as indexed by a comparison with normative expected events, was generally high, but a differential pattern of results emerged across the 3 studies.

Although the average number of events observed in study 3 corresponded to the social normative expected number of 32 events (*d*=0.05), the overall number of eating events logged in studies 1 and 2 were significantly below the social normative threshold. In study 1, the participants missed an average of 3.8 eating events (*d*=0.40) across the 8-day assessment period; in study 2, the rate was even higher at 5.3 missing events (*d*=0.71).

Converting these results into practical significance is not unequivocally possible as *ground truth* (eg, [52-54]) is virtually unavailable (only 2 studies used doubly labeled water as an intake criterion, [6,55]). Multiple criteria have been suggested for evaluating the practical significance of observed effects [56] (see also [20]). Regarding benchmark values, Cohen effect sizes for the overall missing event rates are in the small-to-medium range [57]. Furthermore, the comparison of the effects size values of this study with previous research is limited because of a lack of research. Specifically, the vast majority of EMA studies used signal-contingent data collection protocols in which the participants are prompted (often many times per day) to provide information, and adherence can be defined as the percentage of prompts (eg, signals and reminders) to which the participants responded [23,24,50]. Silvia et al [58] estimated that signal-contingent EMA studies commonly have 15% to 35% prompt-wise missing data rates [59]. Schembre et al [24] reported a mean nonresponse rate of 21% for 10 signal-contingent diet EMA studies, which is similar to the review results from Heron et al [22], who found an overall nonresponse rate of 24%. For the studies in this paper, a comparison of the average observed logging rate over the 8 days to a social normative threshold value of 32 yielded overall missing event rates of 12% (study 1), 17% (study 2), and 1.4% (study 3). However, the social normative threshold of 32 eating events across an 8-day study period and the comparison with signal-contingent EMA studies is clearly debatable and should therefore be interpreted with great caution.

Importantly, the overall higher number of reported eating events in study 3 compared with studies 1 and 2 was also reflected at the level of specific meals. For breakfasts, lunches, and dinners, the participants in study 3 reported an average of almost 1 event per day and between 0.64 and 1.16 more breakfasts, lunches, and dinners than participants in studies 1 and 2. The reasons for the higher momentary adherence rate in study 3 could include individual differences, the prompting strategy used, and technological features.

#### The Impact of Technical Features on Missing Events: Addenda, Prompts, and Skipped Meals

One technological feature that might have increased the number of logged eating events in study 3 is the addendum feature. Eldridge et al [17] reviewed 43 technology-based dietary assessment tools and found that more than 40% included an option to add missing foods, making it the most common customized feature. In the context of paper-and-pencil methods, backfilling is seen as an obstacle to valid assessments. However, as mobile device data are time-stamped, addendum features offer the advantage of providing more insight into adherence, and their added flexibility may also contribute to the participants’ motivation [60,61]. Supporting the notion that addenda substantially contribute to a lower number of missing events, 11% of the complete eating events in study 3 were actually belated.

Furthermore, study 3 included 2 fixed daily reminders, which might have also contributed to a higher logging rate. Reminders and prompts are key features of most EMA and mobile assessment studies for enhancing engagement and adherence. Prompts such as push notifications can now utilize individuals’ contexts to determine the most opportune times to send prompts [62]. *Interruptibility* research has emerged within the field of human-computer interactions, along with text-messaging interventions in psychology and public health [63]. However, current findings from experimental studies indicate that although prompts may encourage greater exposure to message or intervention contents without deterring engagement, they do not always enhance their use [63,64]. Morrison et al compared intelligent notifications, daily notifications within predefined time frames, and occasional notifications within predefined time frames in a stress management intervention and found generally low response rates but a small-to-medium effect on viewed and actioned notifications for the first two compared with occasional notifications. Comparisons between study 1, which did not include any reminder or prompts, and study 2, which included a fixed daily reminder to log one’s meals, supports the notion that predefined reminders might not per se increase momentary adherence. However, in study 3, 2 fixed daily reminders were added, which might have contributed to the higher momentary adherence rate. In particular, the (self-selected) fixed reminder in the evening might have positively impacted momentary adherence because it additionally reminded participants to log eating events they missed logging or skipped during the day. Further supporting this notion, fewer participants in study 3 reported that they were not aware that they had missed logging an eating event compared with studies 1 and 2.

Study 3 also included a technical feature, which allowed the participants to log a skipped meal, and this was used at least once by 78% of the 110 participants. Including the number of skipped meals increased the number of logged eating events by an average of 2 logs across the 8-day study period, which represents 7% of the total entries. Interestingly, skipped meals were reported with almost equal frequency for all three main meals. Pendergast et al [65] assessed skipped meals in young adults in a 4-day EMA study on the following day and found that almost 50% of the participants were regular meal skippers (skipping ≥25% of main meals), with 15% of the sample defined as breakfast skippers, 12% as lunch skippers, and 10% as dinner skippers. Therefore, meal skipping occurs across a broad range of people and affects all main meal types. Assessing skipped meals offers the possibility of increasing measurement precision, as it allows differentiation between skipped meals and missing events. Thus, adding a skipped meal recording feature is likely to have a substantial and consistent effect on measurement quality.

#### Momentary Adherence and Missing Events Over Time: Logging Trajectories

Extending previous EMA diet studies, we analyzed the logging trajectories over time to examine whether momentary adherence declines over time. Across all 3 studies, the logging trajectories of reported meals over time showed a significant, albeit small, decline over time. The trend was slightly more pronounced in study 2 (pseudo-R²=0.06) and less pronounced in study 3 (pseudo-R²=0.04), mirroring the previously discussed results for time-aggregated data. The random intercept model was preferred in all 3 studies, indicating that the participants did not differ significantly in their logging rates over time. Although the results consistently indicate that interindividual differences did not systematically affect logging trajectories over time, we found a differential pattern in dependence of the meal type within each study.

The logging trajectories for the main meals (breakfast, lunch, and dinner) showed no decrease in reporting, except for a small-time effect in study 1 for lunch and dinner. This was surprising, given that the intensive nature of diet EMA protocols and the burden on participants has often been viewed as a major contributing factor in both response fatigue [66] and decreases in momentary adherence. Converging with the present results as well as a recent meta-analysis of mobile EMA studies, no significant study duration effects were found on the rate of response to prompts [50,58,67], which emphasizes the need for a more nuanced understanding of the factors that affect momentary adherence.

The significant negative time effect we found for snacks in studies 2 and 3, which was not found for main meals, might indicate underreporting or measurement reactivity [68-70]. Although underreporting means that snacks were consumed but not logged, which constitutes missing events, measurement reactivity describes the effect that repeated assessments draw attention to the monitored behavior, which can identify problematic behavior and induce behavioral changes. Interestingly, the number of reported snacks dropped noticeably between days 1 and 2 (see Figure 1), which is when the attention effect and identification of problematic behavior is theoretically most likely. However, considering the present data, it is not possible to disentangle underreporting (ie, consumed snacks not being reported) and measurement reactivity effects (ie, an actual decrease in snacks consumed).

#### Perceived Missing Events and Reasons Across Studies

Almost all participants acknowledged that they had missed logging at least one eating event during the study period. Interestingly, the *enrichments* in study 3, namely the 2 fixed daily reminders and an addendum feature, did not result in a lower number of participants recalling a missing event compared with the more *basic* study 1 (92% vs 90%), which suggests that people are aware or assume that their reports are incomplete.

However, examining the number of missing events reported shows a lower median number of perceived missing events in study 1 than in study 3, but compared with the social normative expected number of events, perceived events were lower in study 1 and higher in study 3. Therefore, one might speculate that including technical features such as reminders might not only increase the actual logging of eating events but could also increase the awareness of missing events. Perceived missing events in studies 2 and 3 were more likely to be main meals than snacks, which contrasts with the assumption that irregular eating events might be rather more likely to be missed because of attention and memory effects.

Overall, the reported reasons for missing events showed a similar profile across all 3 studies. The most common reasons were engagement in competing activities and technical issues, whereas situational reasons were less important. In the context of multitasking, further examination shows that although completion rates are affected by lapses in attention, resource, and time scarcity also lead to deliberately deciding against recording an eating event. Although customized prompts might be useful for the former, the latter represents a clear limitation for active real-time assessments. Passive assessments of eating, such as automatic sensing through wearables [52-54] circumvent this limitation. As expected, device-related obstacles (specifically not having the device handy for recording) were an important reason for missing events. Situational barriers, specifically practical reasons that rendered recording as neither feasible nor admissible, were also common reasons for missing events. In contrast, social reasons, such as feeling intimidated by taking a picture, were only rarely noted.

### Study Limitations

Although attrition rates were very low in these studies, biases such as recruitment or volunteer bias need to be considered [71]. Specifically, individuals who are willing to participate in a research study are self-selected and presumably highly motivated and health conscious. Research on participation biases in mailed surveys showed that people who are female, older, or with higher education levels are more likely to return postal questionnaires. Furthermore, people with a poorer health status tend to avoid participating in health surveys [72]. Thus, the predominance of females and students among the participants suggests the presence of a *volunteer bias* in these study series, which limits the generalization of the pattern of results to other population groups. Furthermore, our sample sizes were relatively small in comparison with large-scale epidemiological studies. Although research using mobile EMA is demanding, it would be informative to address the issue of missing event rates with larger samples.

Relatedly, individual differences can also systematically impact momentary adherence. Specifically, previous research has shown that women and people with a college degree are more likely to respond to prompts within signal-contingent EMA studies [58,73]. However, the ICCs across the 3 studies indicated that 38% or less of the observed variance was because of individual differences. Thus, differences in logging rates are more pronounced within individuals than between different people. Overall, the present findings suggest that the actual state of the individual, situational context, and technical features of the mobile app seem to have a greater influence on the adherence rates than stable individual differences (see the study by Sun et al [74] for similar conclusions).

An important factor in mobile app research is the duration of the assessment period, which may impact momentary adherence over time. Although assessment duration has received increasing attention in the study of dietary intake and has been addressed in existing assessment guidelines [41], the different fields and studies use different time periods. Similar to these studies, mobile dietary assessment studies commonly use periods ranging from 1 to 7 days [16]. However, longer assessment periods might be necessary for specific research questions, such as the assessment of micronutrients [41] and the evaluation of intervention studies using dietary self-monitoring. Overall, the study findings need to be interpreted within the context of the 8-day assessment period and the generalizability of the findings, and future studies are needed to determine the maximum number of feasible data collection days.

A further limitation regarding the analysis of the numbers of and reasons for perceived missing events assessed by semistructured interviews in studies 1 and 2. It seems reasonable that memory biases, that is, primacy and recency effects, may have impacted recollection. Consistent with this notion, study 3, which provided the opportunity to report missing events on a daily basis, indicated an average of 2 more logs across the 8-day study period.

The strength of this study series regarding the analysis of logging data across time, that is, showing only a small decline in event logging, and the analysis of reasons for missing events, that is competing activities and technical issues. However, this study series is limited with regard to the precise estimation of missing events. Specifically, based on epidemiological data and social eating norms, the number of missed events was estimated in reference to external criteria rather than the objective assessment of the number of event episodes. This limitation has been widely acknowledged and is accentuated in research over longer assessment periods ranging from weeks to months [52-54,75]. One possibility to acquire objective data is to use video and audio recordings throughout the day to objectively identify the number of eating episodes. However, such an approach would raise ethical issues regarding data privacy, involve time, and require resource-intensive annotation efforts from external observers or the participants themselves. Acknowledging these limitations [41], future studies are needed to estimate the frequency of missing events in reference to objective data.

### Conclusions

Using 3 different indicators, missing events were assessed in 3 mobile image-based dietary EMA studies in more than 180 adults, who reported 5473 eating events, including 3803 main meals, 1505 snacks, and 165 afternoon teas. Given the intensive nature of diet EMA protocols, logging trajectories over time were remarkably stable for main meals. The small significant negative time effect for snacks might indicate underreporting or measurement reactivity. Differences in logging rates were more pronounced within individuals than between different persons. Hence, the actual state of the individual and context seem to have a greater influence on adherence rates than stable individual differences. Supporting this notion, study 3, which included an *enriched* app with reminders, addendum option, and the possibility of recording skipped meals, yielded the highest number of recorded meals. Thus, including such customized features can substantially increase the measurement quality. Engagement in competing activities and technical issues were the most frequently named reasons for perceived missing events, whereas situational reasons were less important. The results emphasize the need for a more nuanced understanding of the factors that affect momentary adherence.

## Appendix

Multimedia Appendix 1 Additional results for studies 2 and 3.

## Abbreviations

ANOVA: analysis of variance
EMA: Ecological Momentary Assessment
EPIC: European Prospective Investigation into Cancer and Nutrition
ICC: intraclass correlation
MIDA: mobile image-based dietary assessment
