# Supplementary material for: Occurrence of and Reasons for “Missing Events” in Mobile Dietary Assessments: Results From Three Event-Based Ecological Momentary Assessment Studies
Source: JMIR Mhealth Uhealth. 2020 Oct 14;8(10):e15430. doi: 10.2196/15430 (PMC7593856; doi:10.2196/15430)
Supplement: Multimedia Appendix 1 [file mhealth_v8i10e15430_app1.docx]

Multimedia Appendix Table 1. Reported eating events by self-classified meal type over 8 days and addendum feature condition (study 2, N=35).

|  | Addendum feature enabled (n=18) | Addendum feature  not enabled (n=17) |  |  |  |
| --- | --- | --- | --- | --- | --- |
| Meal type | M (SD) | M (SD) | *t* value | *df* | *P* value |
| Breakfast | 6.94 (1.47) | 6.82 (3.11) | 0.15 | 22.57 | .89 |
| Lunch | 6.28 (1.60) | 5.76 (1.95) | 0.85 | 33 | .40 |
| Dinner | 6.67 (1.94) | 6.71 (1.96) | -0.06 | 33 | .95 |
| Snacks | 6.11 (4.95) | 6.59 (4.80) | -0.29 | 33 | .77 |
| Afternoon tea | 0.72 (1.53) | 0.76 (0.90) | - | - | - |
| Total | 26.72 (7.35) | 26.65 (7.81) | 0.03 | 33 | .98 |

Multimedia Appendix Table 2. Number of participants by the number of logged snacks across the 8-day study period (study 2, N=35).

|  | Number of participants | | | | | | | |
| --- | --- | --- | --- | --- | --- | --- | --- | --- |
| Number of recorded snacks | Day 1 | Day 2 | Day 3 | Day 4 | Day 5 | Day 6 | Day 7 | Day 8 |
| 0 | 10 | 14 | 19 | 17 | 21 | 18 | 19 | 21 |
| 1 | 9 | 13 | 11 | 15 | 5 | 13 | 7 | 8 |
| 2 | 10 | 6 | 1 | 3 | 8 | 2 | 8 | 5 |
| 3 | 6 | 2 | 4 | 0 | 1 | 0 | 1 | 1 |
| 4 | 0 | 0 | 0 | 0 | 0 | 1 | 0 | 0 |
| 5 | 0 | 0 | 0 | 0 | 0 | 1 | 0 | 0 |

Multimedia Appendix Table 3. Reported and normative expected eating events (without skipped meals) by self-classified meal type over 8 days (study 3, N=110).

| Meal type | Absolute number of meals (%) | M  (SD) | Min./ Max. | Difference observed-normative meals^a^ | *t* value (*df*=109) | Cohen’s *d* | ICC^b^ |
| --- | --- | --- | --- | --- | --- | --- | --- |
| Breakfast | 767 (23.7) | 6.97 (2.64) | 0/23 | -1.03 | -4.08^c^ | 0.39 | .23 |
| Lunch | 670 (20.7) | 6.09 (1.91) | 1/10 | -1.91 | ‑10.46^c^ | 1.00 | .08 |
| Dinner | 787 (24.4) | 7.15 (2.09) | 1/13 | -0.85 | -4.25^c^ | 0.41 | .09 |
| Snacks | 905 (28.0) | 8.23 (5.93) | 0/32 | 0.23 | 0.40 | 0.04 | .34 |
| Afternoon tea | 103 (3.2) | 0.94 (1.08) | 0/5 | - | - | - | .03 |
| Total | 3232 (100) | 29.38 (8.54) | 9/55 | -2.62 | -3.22^d^ | 0.31 | .34 |

^a^ Reference *t*-value was set to a value of 8 meals for individual meal types and to a value of 32 meals for total meals. Negative values indicate fewer observed than normative expected number of meals.

^b^ ICC: intraclass correlation.

^c^ p < .001.

^d^ p < .05.
